# Interaction of Gut Microbiota and Brain Function in Patients With Chronic Insomnia: A Regional Homogeneity Study

**DOI:** 10.3389/fnins.2021.804843

**Published:** 2022-01-05

**Authors:** Ying Feng, Shishun Fu, Cheng Li, Xiaofen Ma, Yunfan Wu, Feng Chen, Guomin Li, Mengchen Liu, Hang Liu, Jiaying Zhu, Zhihong Lan, Guihua Jiang

**Affiliations:** ^1^The Second School of Clinical Medicine, Southern Medical University, Guangzhou, China; ^2^Department of Medical Imaging, Guangdong Second Provincial General Hospital, Guangzhou, China; ^3^Guangdong Traditional Medical and Sports Injury Rehabilitation Research Institute, Guangdong Second Provincial General Hospital, Guangzhou, China; ^4^Department of Medical Imaging, Zhuhai People’s Hospital, Zhuhai, China

**Keywords:** gut microbiota, chronic insomnia, functional magnetic resonance imaging, resting-state, regional homogeneity

## Abstract

Recent studies have shown that the human gut microbiota (GM) plays a critical role in brain function and behavior via the complex microbiome–gut–brain axis. However, knowledge about the underlying relationship between the GM and changes in brain function in patients with chronic insomnia (CI) is still very limited. In this prospective study, 31 CI patients and 30 healthy controls were recruited. Resting-state functional magnetic resonance imaging scans were performed and brain functional alterations in CI patients were evaluated using the regional homogeneity (ReHo) method. We collected fecal samples of CI patients and used 16S rDNA amplicon sequencing to assess the relative abundance (RA) and alpha diversity of the GM. We also performed extensive sleep, mood, and cognitive assessments. Then, we tested for potential associations between the GM profile, ReHo alterations, and neuropsychological changes in CI patients. Our results showed associations between the RA of Lactobacilli, ReHo values in the left fusiform gyrus, and depression scores in CI patients. We also found some bacterial genera related to ReHo values of the right triangular inferior frontal gyrus. In addition, the RA of genus Coprobacter was correlated with ReHo values of the left angular gyrus and with specific cognitive performance. These findings revealed complex relationships between GM, brain function, and behavior in patients with CI.

## Introduction

Chronic insomnia (CI) is one of the most common sleep disorders worldwide, with an estimated prevalence of 10% in the adult population (Morin and Benca, 2012). The condition is defined as subjective complaints of difficulty in initiating or maintaining sleep, or early morning awakening with associated daytime impairments, persisting over a period of 3 months (American Psychiatric Association [APA], 2014; Darien, 2014). CI can lead to mental symptoms such as depression (Baglioni et al., 2011) and can cause daytime cognitive impairments (Fortier-Brochu et al., 2012) such as reduced attention, decreased memory, and executive dysfunction. In the US alone, the cost of treating insomnia is estimated to exceed $20 billion, with total insomnia-related costs exceeding $100 billion per year, which causes a pronounced socioeconomic burden (Wickwire et al., 2016). Previous studies have proposed several models to explain the etiology and pathophysiology of insomnia, including hyperarousal, genetic vulnerability, and specific molecular and cellular mechanisms, but there is still no universally accepted model (Levenson et al., 2015). It is therefore important to investigate potential neuropathological mechanisms in CI and find a novel adjuvant therapeutic method.

In recent years, interest has surged regarding the potential role of the gut microbiota (GM) in the complex microbiome–gut–brain axis (MGBA). Within this axis, GM can modulate emotional behavior and cognitive function through several mechanistic pathways including the immunoregulatory pathway, the neuroendocrine pathway, the vagus nerve pathway, and activation of the hypothalamic–pituitary–adrenal (HPA) axis (Cryan and Dinan, 2012; Foster et al., 2016; Rogers et al., 2016; Sharon et al., 2016). In fact, GM has recently been associated with a series of neurodegenerative brain diseases including Alzheimer’s disease, Parkinson’s disease and amyotrophic lateral sclerosis (Spielman et al., 2018). Furthermore, in the context of neurodegenerative processes, orexin (a peptide also involved in the regulation of the sleep-wake cycle and mood, in eating behavior, in energy homeostasis and in cognitive processes) seems to play an important role, with mechanisms which might involve GM (Mogavero et al., 2021). Recently, several preclinical and clinical studies have identified a close potential relationship between gastrointestinal microbial perturbations and insomnia (Liu et al., 2019; Li Y. et al., 2020; Wang et al., 2020). For example, a study combining high-throughput sequencing and bioinformatic analysis found that the structural composition, signaling pathways, and metabolic function of the GM in insomnia patients were significantly perturbed compared with those of healthy controls (HCs). Additionally, the relative abundance (RA) of the GM at the phylum level was significantly correlated with clinical sleep parameters (Liu et al., 2019). Moreover, one recent study (Li Y. et al., 2020) found that the RA of signature bacteria such as Faecalibacterium and Blautia in CI patients was significantly correlated with self-reported sleep quality and inflammatory cytokines. The evidence suggests that the GM may be an integral contributor to the pathogenesis of insomnia. Despite these findings, there is still a significant gap in establishing a correlation between the GM and the brain in the condition of CI. However, combining advanced neuroimaging techniques with analysis of the GM has tremendous potential to enhance understanding of this connection (De Santis et al., 2019).

Emerging evidence supports the connection between structural or functional aberrance in the brain and the GM profile. For example, using the fractional amplitude of low-frequency fluctuations (fALFF) method, Liu et al. (2021) found a potential relationship between the RA of certain bacteria, changes in fALFF values in specific brain regions, and cognitive function in patients with amnestic mild cognitive impairment. Wang et al. (2019) found that patients with end-stage renal disease displayed complex associations between GM alterations, systemic inflammation, disrupted topology architecture of the default mode network (DMN), and cognitive impairment. In addition, one recent study (Li et al., 2021) combining voxel-based morphometry analysis and regional homogeneity (ReHo) analysis found that the alpha diversity of the GM was correlated with the values of both gray matter volume and ReHo in patients with schizophrenia. Recent clinical studies have revealed the potential association between the GM and insomnia. However, few studies have incorporated neuroimaging techniques, and the potential relationship between the GM and changes in brain function with CI remains unclear.

Advanced resting-state functional magnetic resonance imaging (rs-fMRI) analysis has become a widely used technique for exploring abnormal functional changes of the brain. The combination of rs-fMRI analysis and GM analysis can explore correlations between GM and brain activity in CI and further construct a GM-brain function-behavior model. The ReHo method was found to be reliable and effective in evaluating the level of spontaneous regional activity across the whole brain (Zang et al., 2004). The method has been successfully applied in various neuropsychological disorders including insomnia due to its favorable test-retest reliability and ease of data processing (Kiviniemi, 2008). Several previous studies (Dai et al., 2014; Wang et al., 2016) have found that CI patients showed abnormal ReHo indexes in several brain areas such as the insula, cingulate gyrus, and fusiform gyrus. These altered ReHo values were correlated with sleep quality and psychological scores, which suggests that abnormal ReHo in specific areas may serve as an early biomarker for brain activity changes in CI patients. However, research on the relationship between regional brain function activity and GM composition in the context of CI is still in its infancy.

Our study is the first to explore the relationship between the ReHo alterations and GM composition in CI patients, which may provide objective neuroimaging evidence for a potential neuropathological mechanism of the MGBA involved in CI. In this study, we hypothesized that regional neural activity changes of the brain are associated with the GM profile of CI patients. We aimed to leverage ReHo analysis based on rs-fMRI to investigate brain functional alterations in CI patients and further reveal the underlying relationships between GM profile, ReHo alterations, and neuropsychological changes.

## Materials and Methods

### Participants

A total of 61 subjects were recruited, including 31 CI patients from the Department of Neurology of Guangdong Second Provincial General Hospital, and 30 age-, gender-, and education level-matched HCs recruited from the local community by means of advertisements. All participants were right-handed according to the Edinburgh handedness inventory (Oldfield, 1971) and aged 18–60 years. This prospective study was approved by the Ethics Committee of the Guangdong Second Provincial General Hospital. Informed written consent was obtained from all participants or their legally authorized caregivers.

The diagnosis of CI was determined by two neurologists with 15 years of experience each, based on criteria of the Diagnostic and Statistical Manual of Mental Disorders, version 5 (DSM-V) (American Psychiatric Association [APA], 2014). The neurologists used a semi-standardized psychiatric and sleep-related interview to screen patients. Subjects chosen met the following criteria: (a) self-reported difficulty falling asleep or maintaining sleep, or early morning awakening; (b) difficulty sleeping at least three times per week over a period of 3 months; (c) at least one related daytime impairment (e.g., fatigue, mood disturbance, or impaired cognitive performance); (d) no other sleep disorders (e.g., obstructive sleep apnea or sleep-related movement disorder), serious organic diseases, or mental disorders such as depression or generalized anxiety determined by a semi-standardized psychiatric interview conducted by an experienced clinical psychiatrist with 5 years of experience in clinical psychiatry. In addition, all patients chosen were free of any psychoactive medications for at least 2 weeks prior to and during the study to eliminate drug effects.

CI patients were excluded from the study if they (a) had an abnormal signal in any region of the brain as verified by conventional T1- or T2-weighted fluid-attenuated inversion recovery (FLAIR) MRI; (b) had a history of severe malnutrition, infection, digestive problems such as obvious diarrhea, constipation, irritable bowel syndrome, or inflammatory bowel disease; (c) had a history of drug abuse, alcohol addiction, or regular smoking; (d) had a diet including spicy stimulating food, or took antibiotics, probiotics, prebiotics, or cathartic drugs within 2 months prior to fecal sample collection; (e) were pregnant, nursing, or menstruating females. Inclusion criteria for the HC subjects were as follows: (a) good sleep quality, with an Insomnia Severity Index (ISI) score of <7; (b) no brain lesions or prior substantial head trauma as verified by conventional T1- or T2-weighted FLAIR MRI; (c) no history of psychiatric or neurological diseases; (d) not pregnant, nursing, or menstruating if female.

### Assessment of Sleep Quality, Mental Status, and Cognitive Ability

Prior to MRI scanning, each participant was asked to undergo a battery of standardized neuropsychological assessments. These tests included the Pittsburgh Sleep Quality Index (PSQI) and the Insomnia Severity Index (ISI) to evaluate sleep quality, the Self-Rating Depression Scale (SDS) and Self-Rating Anxiety Scale (SAS) to evaluate mental status, and the Montreal Cognitive Assessment Scale (MoCA) (Nasreddine et al., 2005) to measure global cognitive function. Information processing speed was evaluated using the Digit Symbol Substitution Test (DSST), and working memory was determined by the Digit Span Test (DST). Executive function and attention were evaluated with Trail-Making Test (TMT) A and B. The demographic and neuropsychological details for the included subjects are shown in Table 1.

**TABLE 1:** Demographics and behavioral assessments between CI patients and healthy controls groups.

	CI (*n* = 31)	HC (*n* = 30)	Statistics	*p*-value
**Demographics**				
Age (y)	44.81 ± 11.67	42.27 ± 9.91	0.915	0.364^c^
Gender (F/M)	8/23	10/20	0.415	0.519*^b^*
Education (y)	15.00 (12.00 ∼ 16.00)	16.00 (14.25 ∼ 16.00)	–1.839	0.066^a^
BMI (kg/m^2^)	21.69 ± 1.89	20.99 ± 2.07	1.388	0.170^c^
FD (mm)	0.087 ± 0.043	0.078 ± 0.042	0.83	0.409^c^
**Behavioral assessments**				
PSQI (score)	13.87 ± 2.09	5.23 ± 2.06	16.228	<0.001^c^
ISI (score)	19.39 ± 3.59	5.20 ± 2.07	18.802	<0.001^c^
SAS (score)	52.35 ± 11.00	43.47 ± 6.36	3.848	<0.001^c^
SDS (score)	52.42 ± 7.37	43.57 ± 6.04	5.122	<0.001^c^
TMTA (*s*)	60.74 ± 20.70	42.50 ± 10.11	4.351	<0.001^c^
TMTB (*s*)	137.00 (125.00 ∼ 200.00)	97.00 (77.25 ∼ 113.50)	4.712	<0.001^a^
DSST (*n*)	40.94 ± 15.29	62.07 ± 7.05	–6.895	<0.001^c^
DST (*n*)	12.00 (12.00 ∼ 13.00)	14.00 (13.00 ∼ 15.00)	–3.657	<0.001^a^
MoCA (score)	23.00 (21.00 ∼ 24.00)	28.00 (27.00 ∼ 29.00)	–6.460	<0.001^a^

*^a^Mann–Whitney U test; ^b^χ^2^ test; ^c^independent two sample t-test.*

*Clinical insomnia; DSST, Digit Symbol Substitution Test; DST, Digit Span Test; HC, healthy control; ISI, Insomnia Severity Index; MoCA, Montreal Cognitive Assessment; n, number; PSQI, Pittsburgh Sleep Quality Index; SAS, Self-Rating Anxiety Scale; SDS, Self-Rating Depression Scale; TMTA, Trail Making Test A; TMTB, Trail Making Test B; y, year.*

### Fecal Sample Collection and Gut Microbiota Analysis

Fecal samples of CI patients were collected in sterile containers and immediately stored in a −80°C freezer prior to further processing. First, microbial genome DNA samples were extracted from stool samples using the QIAamp DNA Mini Kit (QIAGEN, Hilden, Germany) according to the manufacturer’s instructions. After evaluating DNA quality and quantity, a specific hypervariable region of 16s rDNA was selected for PCR amplification based on the MiSeq Illumina platform and following the Illumina protocol. Next, the raw reads were filtered to remove adaptors and low-quality and ambiguous bases. Based on the overlapping relationships between the reads, the paired reads were assembled into a sequence to obtain tags of the hypervariable region, using the Fast Length Adjustment of Short reads program (FLASH, v1.2.11) (Magoč and Salzberg, 2011). Reads without overlapping relationships were removed. Tags with primers for specific fragment amplification were processed using CUTADAPT (Martin, 2011), and chimeras in the tags were removed by the UCHIME method in VSEARCH (v2.3.4) (Rognes et al., 2016). Remaining optimized spliced sequences were stepwise clustered into operational taxonomic units (OTUs) at a 97% similarity threshold using the UPARSE algorithm. These OTU representative sequences were mapped to the database [Silva: v128 (Quast et al., 2013); Greengene: Release13.8 (DeSantis et al., 2006); UNITE: Release 5.0 (Abarenkov et al., 2010)] for species annotation and classification using the Ribosomal Database Project (RDP) Classifier (v2.2) (Wang et al., 2007) with the confidence threshold set to 0.8. The RA of OTU indicates the species richness of the samples. Alpha diversity measures including the Chao index, Ace index, Shannon index, and Simpson index were also calculated using MOTHUR (v1.31.2) (Schloss et al., 2009).

### Magnetic Resonance Imaging Data Acquisition

All participants were scanned using a 3.0-T MRI scanner (Ingenia; Philips, Best, Netherlands) at the Department of Medical Imaging at Guangdong Second Provincial General Hospital. Each subject wore ear plugs to minimize scanner noise and was positioned supine with the head snugly secured by a belt and foam pads to reduce head movements. During rs-fMRI data acquisition, all participants were instructed to lie quietly with their eyes closed but to try not to fall asleep or think of anything specific while inside the scanner. The rs-fMRI datasets were obtained using a gradient-echo planar imaging (EPI) sequence with the following parameters: repetition time (TR) = 2,000 ms; echo time (TE) = 30 ms; matrix = 64 × 61; field-of-view (FOV) = 224 mm × 224 mm; flip angle (FA) = 90°; slice thickness = 3.5 mm with a 1.0 mm gap; interleaved scanning. For each participant, 33 transverse slices covering the whole brain at all 240 volumes were acquired over approximately 8 min. In addition, high-resolution anatomical images were acquired using a T1-weighted 3D gradient-echo sequence with the following parameters: 185 axial slices, TR = 7.9 ms, TE = 3.6 ms, FA = 8°, slice thickness = 1.0 mm, no gap, matrix = 256 × 256, and FOV = 256 mm × 256 mm. We also obtained the T1-weighted images and T2-FLAIR images to detect brain lesions. Subsequently, some questions were asked to evaluate the cooperation status of the subjects.

### Data Processing and Regional Homogeneity Calculations

The rs-fMRI datasets were preprocessed using DPARSF 4.3 Advanced Edition^1^. The first 10 time points for each participant were discarded to allow the signal to reach equilibrium and the subjects to adapt to the scanning environment. The remaining rs-fMRI datasets were then corrected for intra-volume acquisition time delay and inter-volume head motion. To minimize the influence of head motion, data from all participants with head motion >1.0 mm in any direction or 1.0° of any angular dimension and volume-to-volume mean frame-wise displacement (FD) > 2.0 mm (Power et al., 2015) were excluded. Individual T1 structural images were segmented (white matter, gray matter, and cerebrospinal fluid) using the Segmentation toolbox, and the DARTEL toolbox was used to create a study-specific template for accurate normalization. Subsequently, the functional images were co-registered to the structural images and normalized into standard Montreal Neurological Institute (MNI) space with an isotropic voxel size 3 mm × 3 mm × 3 mm. Several covariates and their temporal derivatives were then regressed out from the time course of each voxel, including the brain’s global mean, white matter, and cerebrospinal fluid signals, as well as the Friston 24 parameters of head motion (Friston et al., 1996). To reduce the effects of low-frequency drift and high-frequency noise, the data were further detrended and temporally band-pass filtered (0.01–0.1 Hz).

The ReHo calculation procedure was the same as those reported in previous studies (Zang et al., 2004). Briefly, the ReHo index was estimated by calculating Kendall’s coefficient of concordance (KCC) of the time series of a given voxel with those of its 26 nearest neighbors in voxel-wise way. The ReHo index indicated the degree of regional temporal synchronization of the cluster with a given voxel in the center. For optimization, each ReHo map was divided by its averaged KCC value for the whole brain. Then, the standardized ReHo map was spatially smoothed with a 12-mm full-width at half-maximum Gaussian kernel to reduce noise and residual differences in gyral anatomy.

### Statistical Analysis

Statistical analyses were performed using the SPSS software package version 25.0 (SPSS Inc., Chicago, IL, United States). A two-sample *t*-test was used to compare the continuous variables and an χ^2^ test was used to compare the qualitative variables of gender. Before the analyses, the Shapiro–Wilk test was used to test for normality of quantitative data. Normally distributed data were presented as mean ± standard deviation and then assessed by independent two-sample *t*-test. Mann–Whitney *U* tests were used to analyze the data with non-normal distribution, which were reported as median and inter-quartile range.

To explore the ReHo differences between CI patients and HC subjects, a two-sample *t*-test was performed on the individually smoothed ReHo maps, taking age, gender, education level, body mass index (BMI), and mean FD as covariates. Benjamini–Hochberg false discovery rate (FDR) correction (Benjamini and Hochberg, 1995) was performed to address the multiple comparisons at a significance level of 0.05 (*p* < 0.05). Furthermore, the mean ReHo values of significant areas were extracted independently using the Resting-State fMRI Data Analysis Toolkit^2^. To further investigate the relationship among the factors derived from the gut microbiota, brain function and clinical variables, we selected several GM features including alpha diversity and specific gut taxa such as Bacilli, *Lactobacillus*, *Erysipelatoclostridium*, Tyzzerella 4, *Ruminococcus gnavus* group, and Coprobacter which have been reported to be associated with CI and other psychiatric disorders (Poroyko et al., 2016; Li et al., 2018; Aizawa et al., 2019; Ma et al., 2019; Vindegaard et al., 2020). Correlations between GM parameters (RA and alpha diversity), neuropsychological assessment scores, and ReHo values in significantly different areas were performed using partial correlation analysis, controlling for age, gender, education level, BMI, and mean FD. Bonferroni correction was carried out in correlation analyses for multiple comparisons. Clustering analysis based on correlation coefficient and graphic representation were performed using the pheatmap and ggplot2 packages of RStudio software (version 1.4.1106^3^). All statistical tests were two-sided, and differences in values of *p* < 0.05 were considered statistically significant.

## Results

### Demographic and Neuropsychological Results

There were no significant inter-group differences in terms of age, gender, education level or BMI (all *p* > 0.05). As expected, in neuropsychological assessments, there were significant differences (*p* < 0.05) between CI patients and HC subjects in all of the sleep measures (PSQI and ISI), mood measures (SAS and SDS), and cognitive measures (TMTA, TMTB, DSST, DST, and MoCA). Demographic and neuropsychological assessment results are shown in Table 1. Notably, CI patients were found to score distinctly lower in each mental and cognitive assessment than HC subjects (*p* < 0.001).

### Altered Regional Homogeneity in CI Patients

**FIGURE 1:**
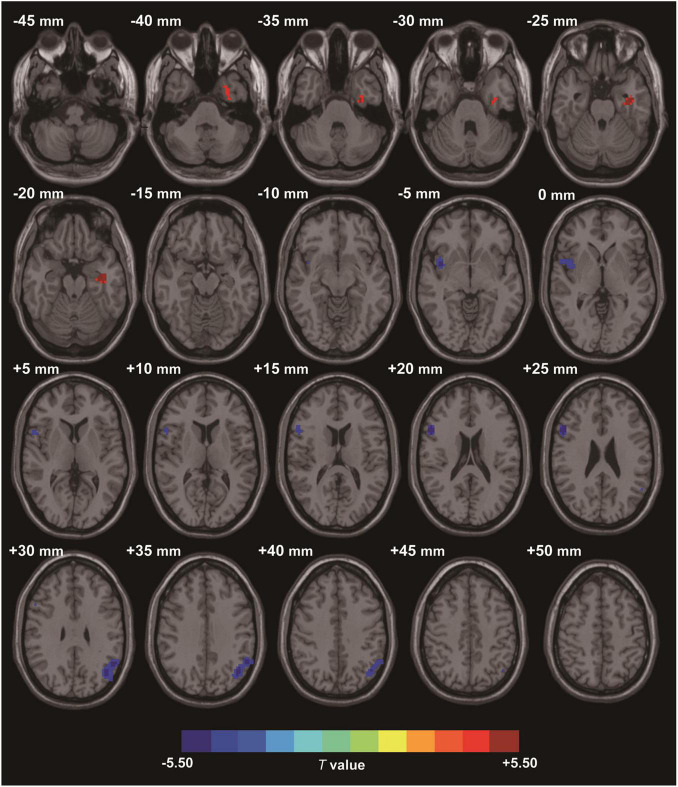
Clusters showing statistically significant different ReHo values in patients with CI compared with HCs (*p* < 0.05, FDR corrected). CI patients showed increased ReHo values in the left fusiform gyrus (warm colors), as well as decreased ReHo values in the right triangular inferior frontal gyrus and left angular gyrus (cold colors). AT- score bar is shown below. Red and green denote increases and decreases in ReHo, respectively.

Compared to the HC group, the data showed that CI patients had significantly greater ReHo values in the left fusiform gyrus (LFG). Furthermore, we detected decreased ReHo values in the left angular gyrus (LAG) and the right triangular part of inferior frontal gyrus (IFG) that extended to the right insula (Table 2 and Figure 1) in CI patients when compared with the HC group.

**TABLE 2:** Brain regions with abnormal ReHo in CI patients compared with healthy controls.

Brain area	Voxel size	Peak MNI coordinate			*T*-value	*p*-value
		*x*	*y*	*z*		
Fusiform_L	77	−39	−12	−21	4.862	<0.001
Frontal_Inf_Tri_R	132	54	18	24	–5.317	<0.001
Angular_L	172	−54	−54	33	–4.967	<0.001

*L, left; R, right; MNI, Montreal Neurological Institute; ReHo, regional homogeneity.*

### Associations Between Gut Microbiome, Regional Homogeneity Values in Abnormal Regions, and Neuropsychological Assessments

**FIGURE 2:**
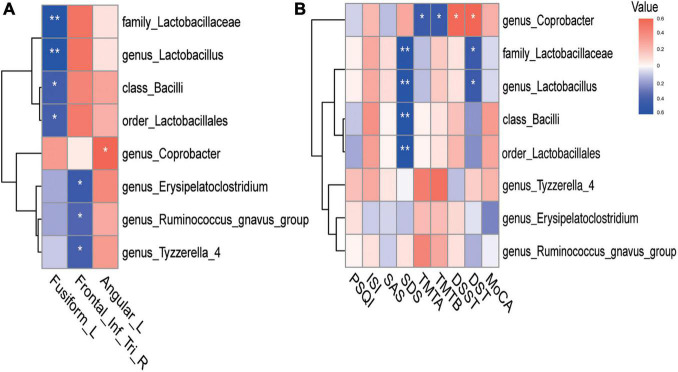
Heatmap of the associations between GM and ReHo values in abnormal regions and neuropsychological assessments in CI patients. **(A)** The associations between GM and ReHo values in abnormal regions. **(B)** The associations between GM and neuropsychological assessments. The Heat map shows the correlation coefficients between these variables, where the intensity of the color [positive (blue) or negative (red)] is proportional to the strength of the association. *p < 0.05, **p < 0.01. GM, gut microbiota; ReHo, regional homogeneity.

We subsequently explored the potential relationships between GM parameters (RA and alpha diversity), ReHo values with group differences, and neuropsychological test scores in CI patients. The heat map of GM, ReHo measures, and neuropsychological assessments was constructed based on significant correlation coefficients (*p* < 0.05). Analysis revealed a variety of significant correlations between ReHo alterations and GM composition (Figure 2A). For instance, we observed that increased ReHo values of the LFG were negatively correlated with RA of the class Bacilli (*r* = −0.416, *p* = 0.020), order Lactobacillales (*r* = −0.408, *p* = 0.023), family Lactobacillaceae (*r* = −0.549, *p* = 0.001), genus *Lactobacillus* (*r* = −0.549, *p* = 0.001). After the strict Bonferroni correction, family Lactobacillaceae (*r* = −0.549, *p* = 0.004) and genus *Lactobacillus* (*r* = −0.549, *p* = 0.004) still showed significant negative correlations with ReHo values in the LFG (Supplementary Figure 1A). We also found that the decreased ReHo values of the right triangular part of IFG were negatively correlated with RA of the genus *Erysipelatoclostridium* (*r* = −0.449, *p* = 0.011), genus Tyzzerella 4 (*r* = −0.409, *p* = 0.022), and genus *Ruminococcus gnavus* group (*r* = −0.374, *p* = 0.038). Notably, the correlation between the genus *Erysipelatoclostridium* (*r* = −0.449, *p* = 0.034) and ReHo values of the right triangular IFG was still significant after the strict Bonferroni correction (Supplementary Figure 1B). In addition, the decreased ReHo values of the LAG were positively correlated with RA of the genus Coprobacter (*r* = 0.390, *p* = 0.030). There was no significant correlation between ReHo values in abnormal regions and alpha diversity index of the GM.

For microbiomes showing correlations with ReHo values, correlation analyses were further performed to evaluate their associations with clinical variables. Figure 2B illustrates that changes in emotion and cognition of CI patients showed several correlations with specific microbial traits. For example, depression scores (SDS) showed strong negative correlations with RA of the class Bacilli (*r* = −0.500, *p* = 0.017), order Lactobacillales (*r* = −0.495, *p* = 0.019), family Lactobacillaceae (*r* = −0.484, *p* = 0.023), and genus *Lactobacillus* (*r* = −0.484, *p* = 0.023), after Bonferroni correction (Supplementary Figures 2A–D). Poor cognitive scores in TMTA (*r* = −0.406, *p* = 0.023) and TMTB (*r* = −0.410, *p* = 0.022) tests showed strong negative correlations with RA of the genus Coprobacter. Better cognitive performance in DSST (*r* = 0.370, *p* = 0.040) and DST (*r* = 0.384, *p* = 0.033) tests were positively associated with RA of the genus Coprobacter. There were no significant associations between RA of the genus Coprobacter and these cognitive assessments after the Bonferroni correction.

**FIGURE 3:**
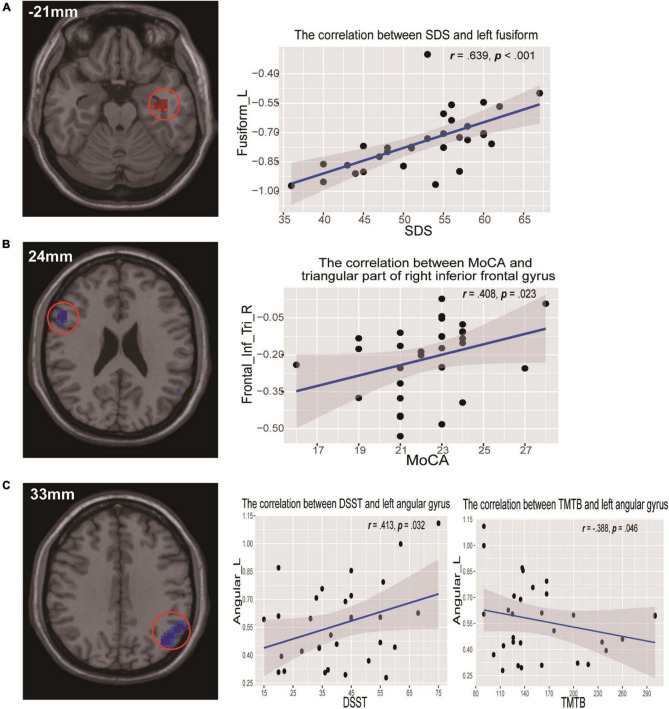
Scatterplots show the relationships between ReHo values and neuropsychological assessments in participants with CI. **(A)** ReHo values of the left fusiform gyrus were significantly positively correlated with SDS scores. **(B)** ReHo values of the right triangular inferior frontal gyrus were positively correlated with MoCA scores. **(C)** ReHo values of the left angular gyrus were positively correlated with DSST scores but negatively correlated with TMTB scores. DSST, Digit Symbol Substitution test; MoCA, Montreal Cognitive Assessment; ReHo, regional homogeneity; SDS, self-rating depression scale; TMTB, Trail Making Test B.

In addition, for correlation analyses between ReHo values and neuropsychological assessments in CI patients, we found that ReHo values for the LFG showed a significant positive correlation with SDS scores (*r* = 0.639, *p* = 0.000) (Figure 3A). Decreased ReHo values of the right triangular part of the IFG were positively correlated with MoCA scores (*r* = 0.408, *p* = 0.023) (Figure 3B). There were positive correlations between decreased ReHo values of the LAG and DSST scores (*r* = 0.413, *p* = 0.032), and a negative correlation between TMTB scores (*r* = −0.388, *p* = 0.046) and ReHo values in the same area (Figure 3C). There were no significant correlations between ReHo values in these abnormal regions and sleep quality parameters (PSQI and ISI).

## Discussion

To the best of our knowledge, this is the first study to explore whether resting-state regional functional homogeneity modulated by CI might be associated with microbial composition in patients with CI. The results revealed that CI patients revealed complex associations between the RA of specific gut taxa, abnormal regional neuronal activity (ReHo values), and corresponding neuropsychological changes. In particular, the data showed a correlation between the RA of Lactobacilli, ReHo values in the LFG, and SDS scores in CI patients. We also identified a relationship between some bacterial genera and the ReHo values of the right triangular IFG. In addition, the RA of genus Coprobacter was correlated with ReHo values of the LAG, as well as with specific cognitive performance. These findings may provide neuroimaging evidence to support the association between the GM and functional abnormalities of the brain in CI.

First, our exploratory analyses observed the relationships between Lactobacilli RA at levels from order to genus, ReHo values of the LFG, and SDS scores in CI patients. Our MRI analysis found increased ReHo values of the LFG in CI patients. The fusiform gyrus is known to be mainly involved in visual processing, face perception, and emotional perception processing, especially in emotional expression recognition (Groenewold et al., 2013; Bae et al., 2019; Li et al., 2019). Our findings were consistent with previous rs-fMRI studies in patients with insomnia (Dai et al., 2014; Li et al., 2016; Wang et al., 2016), which also found regional functional abnormalities in the fusiform region. In previous studies on depression, functional abnormality in the fusiform was also detected and positively correlated with the degree of depression (Wu et al., 2011; Wang et al., 2012; Gong et al., 2020). In the present study, the ReHo values in the LFG were positively associated with SDS scores, which further suggested that this area of the brain may play an important role in emotional regulation in patients with CI. Most interesting were the significant negative associations between Lactobacilli RA and ReHo values in the LFG in our study. *Lactobacillus*, as a probiotic microorganism, has been reported to confer a beneficial impact on physical and mental health. Many clinical trials have found that probiotic interventions could significantly ameliorate anxiety and depression symptoms in human subjects (Messaoudi et al., 2011; Akkasheh et al., 2016; Pinto-Sanchez et al., 2017). Our findings of a negative association between depression scores in CI and Lactobacilli RA further support the idea that Lactobacilli may play a role in regulating mood. Moreover, the RA of Lactobacilli was negatively associated with abnormal functional changes in the LFG, which may provide further insights on the relationship between Lactobacilli and brain function in patients with CI. Of interest, in previous studies, associations were also found between the *Lactobacillus* and functional connectivity changes within the resting-state brain network that included affective regions (Tillisch et al., 2013). However, the exact mechanism underlying the observed correlations is still unclear. It has been reported that *Lactobacillus* may synthesize and release gamma amino butyric acid (GABA) (Wu et al., 2017; Wang et al., 2018) and may also attenuate the response of the HPA axis to stress (Sudo et al., 2004; Bravo et al., 2011). It is speculated that dysregulation of GABA and the HPA axis could play a significant role in the pathogenesis of insomnia (Levenson et al., 2015) or depression (Verduijn et al., 2015). We speculated that these neuroendocrine pathways might mediate the influence of Lactobacilli on the neurological activity of emotion processing in the fusiform area of patients with CI, similar to how the complex MGBA occurs (Cryan and Dinan, 2012). Accordingly, our findings provide preliminary evidence of neural correlations for probiotic intervention as a possible new complementary treatment to improve sleep and mood in CI patients.

We also identified significant negative correlations between some bacterial genera in the gut and ReHo values of the right triangular IFG. The right triangular IFG is a part of the prefrontal cortex (Zhao et al., 2016), which acts as a modulator of higher-order cognitive processes including attention, working memory, and executive function (Lara and Wallis, 2015). This area has frequently been found to be abnormal in patients with CI (Altena et al., 2008; Dai et al., 2016; Pang et al., 2018). In our study, CI patients exhibited decreased ReHo in the right triangular IFG, and the ReHo values were positively correlated with cognitive function (MoCA score), which was lower in CI patients than in HC subjects. Thus, we supposed that chronic lack of sleep can impair the function of the right triangular IFG, which may contribute to cognitive deficits in CI patients. In addition, the association between the RA of some gut bacteria and abnormal ReHo values of the IFG observed in our study may suggest a potential communication mechanism between the GM and brain function in CI patients. Other studies have also detected abnormalities in the prefrontal cortex that were associated with the GM profile. For example, Cai et al. (2021) found significant positive correlations between intra-network connectivity in the lateral prefrontal cortex and GM diversity. Genus *Erysipelatoclostridium*, a taxon known to be associated with inflammation (Xiao et al., 2021), has positive associations with lipopolysaccharide (Li L. L. et al., 2020), an endotoxin that can elicit a strong immune response resulting in central nervous system inflammation (Galland, 2014). Genus *Ruminococcus gnavus*, populations of which bloom in various inflammatory diseases (Henke et al., 2019), has been reported to synthesize and secrete a polysaccharide that induces the production of inflammatory cytokines. Similarly, Tyzzerella_4 has been reported as the potential pathogenic bacteria genera (Fomenky et al., 2018), and its RA level was found to be profoundly increased in chronic inflammatory bowel disease (Qiu et al., 2017; Olaisen et al., 2021). In our results, these pro-inflammatory bacteria were negatively associated with abnormal brain function in CI patients. Interestingly, previous research suggested that systematic inflammation was associated with insomnia symptoms and could be a pathway linking gut microbiome with insomnia (Patel et al., 2009; Jackson et al., 2015; Fernandez-Mendoza et al., 2017). For example, one recent study Li Y. et al. (2020) found that the signature bacteria in CI patients were significantly correlated with insomnia symptoms and elevated levels of inflammatory cytokines. Therefore, we speculated that there was a possible association between pro-inflammatory bacteria and abnormal brain functional activity in CI patients, and that inflammatory pathways may play an important role in this association.

In addition, we discovered associations among decreased ReHo values of the LAG, the RA of genus Coprobacter in the gut, and specific cognitive performance in CI patients. The angular gyrus has been identified as a key parietal node of the DMN (Bluhm et al., 2008) and is involved in a variety of cognitive functions including language and semantic processing, recognition and attention, and episodic memory retrieval (Thakral et al., 2017). Previous resting-state brain function studies have suggested that DMN impairment may be a hallmark of insomnia (Nie et al., 2015; Marques et al., 2017). In addition, we observed that ReHo values of the LAG in CI patients were positively correlated with better scores on DSST and DST tests, which measure the cognitive domains of information processing, psychomotor speed, and working memory (Chung et al., 2020). Moreover, the ReHo values were negatively correlated with worse scores in TMT A and B, which measure the cognitive domains of executive function and attention (Bowie and Harvey, 2006). Accordingly, we speculated that decreased ReHo values for LAG might be involved in the extensive cognitive decline of CI patients. Notably, ReHo values in the LAG were positively associated with the RA of Coprobacter in our study. Genus Coprobacter has been found to be positively associated with insulin supplementation (Jena et al., 2020), an indigestible fiber that can generate short-chain fatty acids (Weitkunat et al., 2015) and improve digestion, memory, and mood in humans (Smith et al., 2015). Our results showed that the RA of this bacteria was positively correlated with better cognitive performance in CI patients, which may indicate potential benefits of this gut bacteria. We also noted a complex relationship between the genus Coprobacter, functional abnormality in a key region of the DMN (angular gyrus), and cognitive impairments in CI patients. There have been prior efforts to explore the associations between GM and DMN abnormalities and cognitive ability in healthy and clinical conditions. Wang et al. (2019) identified a relationship among the GM, DMN dysfunction, and cognitive impairments in patients with end-stage renal disease. Another recent study (Cai et al., 2021) in healthy young adults found that internetwork functional connectivity involving the DMN mediated the relations of GM with sleep quality and executive function. Our study extends these findings by discovering potential associations between the GM, regional brain functional abnormity, and cognitive impairments in CI patients. These findings further suggest that GM-brain function-behavior associations might play a role in the potential neurobiological mechanisms of CI.

### Limitations

Our study had several limitations. First, we did not collect fecal samples from the HC group to detect GM differences between them and CI patients. However, many published studies have explored the relationship between the GM and insomnia, and have detected significant differences in GM composition between CI patients and HCs (Liu et al., 2019; Li Y. et al., 2020). Thus, although we did not measure GM differences between the two groups, the findings of this study should be reliable with regard to patients with CI. Second, this is a preliminary cross-sectional study. Future longitudinal studies with gut flora intervention may help to determine the causal relationship between the GM and the brain. Third, although we recruited all subjects from the same site and used strict exclusion criteria to eliminate the potential effect of factors such as dietary differences or other interventions, we could not eliminate all possible confounding factors affecting GM composition. Fourth, the metabolic and inflammatory changes of intestinal flora should be further studied in order to shed light on the possible underlying mechanisms. Finally, we analyzed only associations between regional brain functional activity and GM composition, and did not study the brain structural changes. Multi-modal neuroimaging combined with structural and functional data may explore the interaction between the GM and the brain more comprehensively.

## Conclusion

Based on rs-fMRI data using a ReHo method, our study found that regional brain functional abnormalities and emotion/cognition related changes had significant correlations with the specific bacterial taxa of GM in CI patients. Our findings may provide insights into the potential role of the GM in the underlying neuropathology of CI patients and could have significant clinical value in targeting the GM as an intervention for treatment of CI patients.

## Data Availability Statement

The raw data supporting the conclusions of this article will be made available by the authors, without undue reservation, to any qualified researcher.

## Ethics Statement

The studies involving human participants were reviewed and approved by Ethics Committee of the Guangdong Second Provincial General Hospital. The patients/participants provided their written informed consent to participate in this study. Written informed consent was obtained from the individual(s) for the publication of any potentially identifiable images or data included in this article.

## Author Contributions

GJ and CL contributed to the design of the study. YF, GL, ML, HL, JZ, and ZL contributed to the data acquisitions. YF, SF, FC, and ZL contributed to the data analysis. YF, SF, XM, YW, and GJ contributed to the results interpretation. YF, SF, CL, and GJ contributed to manuscript preparation. All authors contributed to the manuscript revision and approved the final version of the manuscript.

## Conflict of Interest

The authors declare that the research was conducted in the absence of any commercial or financial relationships that could be construed as a potential conflict of interest.

## Publisher’s Note

All claims expressed in this article are solely those of the authors and do not necessarily represent those of their affiliated organizations, or those of the publisher, the editors and the reviewers. Any product that may be evaluated in this article, or claim that may be made by its manufacturer, is not guaranteed or endorsed by the publisher.

## Footnotes

1
http://rfmri.org/DPARSF

2
http://resting-fmri.sourceforge.net

3
http://www.rstudio.org
