# Supplementary material for: Interaction of Gut Microbiota and Brain Function in Patients With Chronic Insomnia: A Regional Homogeneity Study
Source: Front Neurosci. 2022 Jan 5;15:804843. doi: 10.3389/fnins.2021.804843 (PMC8766814; doi:10.3389/fnins.2021.804843)
Supplement: Supplementary file 1 [file Data_Sheet_1.PDF]

Supplementary Material

1a

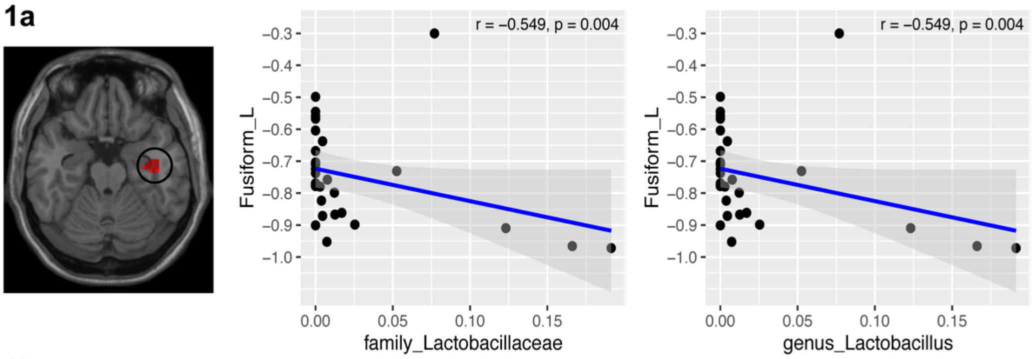

1b

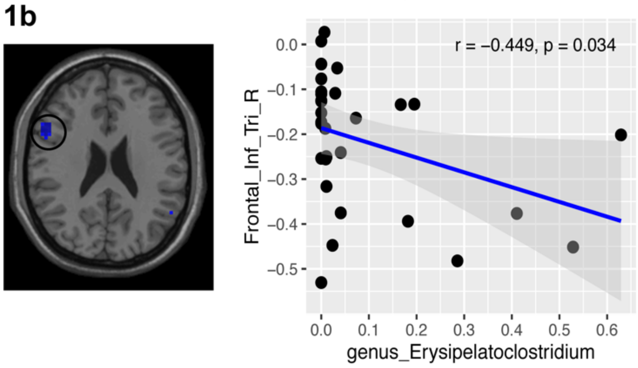

FIGURE 1 | Significant correlations between altered ReHo values and GM in CI group ($p < 0.05$, Bonferroni correction). **(1a)** Scatter plots show negative correlations between ReHo values of the left fusiform gyrus (Fusiform_L) and RA of the GM. **(1b)** Scatter plot shows a correlation between ReHo values of the right triangular inferior frontal gyrus (Frontal_Inf_Tri_R) and RA of the GM. Abbreviations: CI: chronic insomnia; GM: gut microbiota; RA: relative abundance; ReHo: regional homogeneity.

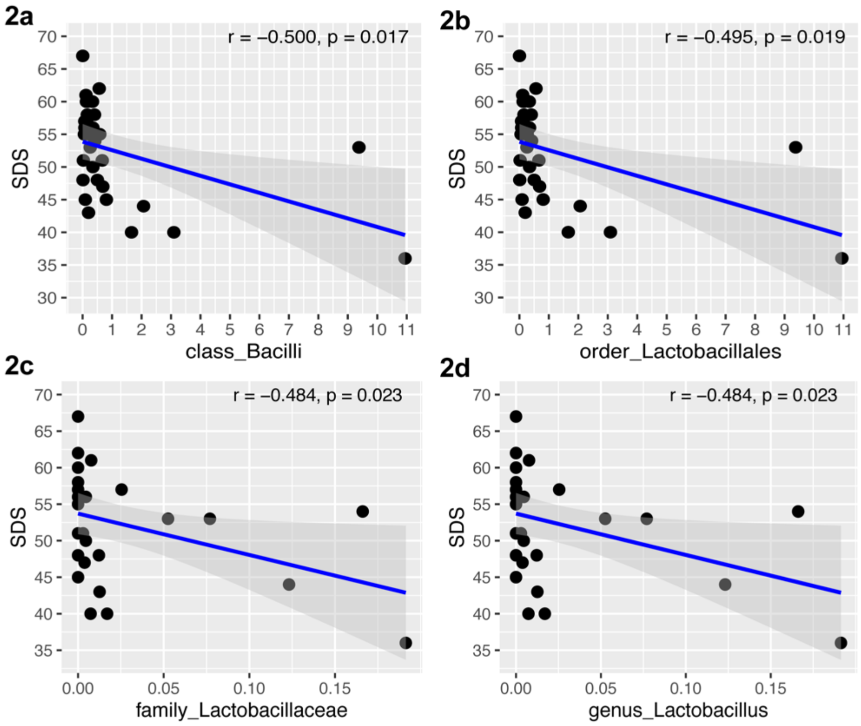

FIGURE 2 | Scatterplots show the relationships between SDS scores and RA of the GM in CI group after the Bonferroni correction. Abbreviations: CI: chronic insomnia; GM: gut microbiota; RA: relative abundance; SDS: Self-rating depression scale.
